# Urotensin II levels in patients with chronic kidney disease and kidney transplants

**DOI:** 10.3109/03009734.2011.626541

**Published:** 2012-02-15

**Authors:** Mehmet Hursitoglu, Tufan Tukek, Mehmet Ali Cikrikcioglu, Osman Kara, Rumeyza Kazancioglu, Oktay Ozkan, Mustafa Cakirca, Fatih Akdogan, Erdal Gundogan, Sengul Aydin, Ismet Beycan, Meltem Gursu, Serkan Dogan, Aybala Erek

**Affiliations:** ^1^Internal Medicine Department, Vakif Gureba Training & Research Hospital, Fatih, Istanbul, Turkey; ^2^Nephrology Clinic, Haseki Training & Research Hospital, Haseki, Istanbul, Turkey; ^3^Nephrology Clinic, Vakif Gureba Training & Research Hospital, Fatih, Istanbul, Turkey; ^4^Microbiology Department, Vakif Gureba Training & Research Hospital, Fatih, Istanbul, Turkey; ^5^Ahenk Laboratory, Istanbul, Turkey; ^6^Biochemistry Department, Vakif Gureba Training & Research Hospital, Fatih, Istanbul, Turkey

**Keywords:** Calcineurin inhibitors, CKD, cyclosporine, kidney transplantation, urotensin II

## Abstract

**Objective.:**

Urotensin II is a potent vasoactive peptide that has been implicated in the pathophysiology of many diseases. There is no study reporting the role and level of this peptide in recipients of kidney transplant. So we aimed to study the plasma levels of urotensin II in this group of patients.

**Methods.:**

Plasma urotensin II levels were analyzed in 110 subjects, who were divided into three groups: group 1 (35 kidney transplant recipients), group 2 (36 patients with chronic kidney disease), and group 3 (39 healthy controls).

**Results.:**

Analysis of logarithmic transformation of urotensin II, i.e. log (urotensin II × 1000) levels, with a one-way analysis of variance yielded a *P* value of 0.001. *Post-hoc* analysis showed significantly higher log (urotensin II × 1000) levels in group 1 than groups 2 and 3 (*P* = 0.001 and 0.017, respectively). One of the important features of the subjects of this group was that they were taking immunosuppressive drugs because of renal transplantation.

**Conclusions.:**

High urotensin II levels in recipients of kidney transplants could be drug-related (immunosuppressive drugs) and may be of practical importance that may be used to improve the long-term outcome of the patients.

## Introduction

Urotensin II (UII) is a potent 11-amino acid vasoactive peptide that produces vasodilatation and inotropic effects in addition to its powerful vasoconstrictive effect. UII acts by binding to a G(q/11) protein-coupled urotensin II receptor (UTR) (1). Recent studies have shown increased expression of UII and its receptors in animals and patients with hypertension, heart failure, atherosclerosis, and diabetic nephropathy (1–4). Thus, UII has been implicated in the pathophysiology of the above-mentioned disorders.

The kidney plays a major role in UII production, which may contribute to its hemodynamic effects (5). UII can also be synthesized in non-renal tissue, such as the heart (6). Some researchers have shown that UII may play a cardioprotective role in patients with ischemic heart disease and chronic renal failure (7,8). Furthermore, several studies have shown increased UII levels in patients with chronic kidney disease (CKD) (1,2,9,10). Kidney transplantation is an important treatment model for CKD, and it is being increasingly employed worldwide (11,12). To our knowledge, no studies have been reported regarding UII levels in patients who have undergone a kidney transplantion. Thus, the purpose of this pilot study was to investigate and compare UII levels among kidney transplant recipients, CKD patients, and normal subjects.

## Materials and methods

This study was approved by our local ethics board, and written informed consent was obtained from all participants. Altogether 110 subjects were enrolled, and they were divided into three groups (Table I). The exclusion criteria were: age <18 years; presence of ischemic heart disease, chronic liver disease, or malignancy; or an inability to provide written consent. The inclusion criteria were as follows: For group 1 (tx) patients: those who had a kidney transplant for more than 4 months and no signs and/or symptoms of any acute or chronic infection or rejection. For group 2 (CKD) patients: those with stages 4 and 5 CKD (13), and in whom dialysis therapy had not been initiated. For group 3: healthy control subjects with normal renal function and no obvious acute or chronic disease.

**Table I.** Characteristics of the participants.

	Group 1	Group 2	Group 3	*P* value
*n*	35/110	36/110	39/110	-
Age (years)	37.4 ± 13.8	36.8 ± 6.8	38.3 ± 5.6	NS
Sex (F/M) (*n*)	12/23	14/22	18/21	NS
Creatinine (RI 0.60–1.10 mg/dL)	1.10 ± 0.20	5.98 ± 1.52	0.75 ± 0.23	0.001^a^, NS^b^, 0.000^c^
CCBs^d^	24/35 (68.6%)	21/36 (58.3%)	0/39 (0.0%)	NS^a^
ACE inhibitors or ARBs^d^	10/35 (28.6%)	14/36 (38.9%)	0/39 (0.0%)	NS^a^
UII (ng/mL)	0.66 (0.16–188)^e^	0.48 (0.06–1.14)^e^	0.59 (0.06–1.85)^e^	0.017^f^

^a^Comparing group 1 with group 2.

^b^Comparing group 1 with group 3.

^c^Comparing group 2 with group 3.

^d^Alone or in combination with other antihypertensive drugs.

^e^Median (min–max).

^f^Kruskal–Wallis test.

RI = reference interval; NS = not significant; CCBs = calcium channel blockers; ACE = angiotensin-converting enzyme; ARBs = angiotensin receptor blockers; UII = plasma urotensin II level.

After obtaining demographic data of the participants, a thorough clinical evaluation and physical examination (including measurements of weight, height, and blood pressure) were performed. After an overnight fast, blood samples (without anticoagulant) for urea and creatinine determination and another one for urotensin II assay (details below) were drawn from the participants. The above-mentioned tests were performed at Ahenk Laboratory (Istanbul, Turkey). Glomerular filtration rate (GFR) was calculated according to the Cockcroft–Gault formula (GFR = [140–age] × weight (kg) / [serum creatinine × 72] × 0.85, if female) (14).

### UII assay

Urotensin II (human) was measured by an enzyme-linked immunoassay (EIA) method (15). A specific and sensitive EIA kit was used for this assay (Phoenix Pharmaceutical Inc., California, USA). The intra- and inter-assay coefficients of variations were <15% and <5%, respectively. The minimum detectable concentration was 0.06 ng/mL. There was no cross-reactivity with endothelin-1, angiotensin II, PAMP-20, I-ANP-28, bradykinin, and neurotensin, but there was <15.7% cross-reactivity with UII-related peptides. Blood samples were collected into Lavender Vacutainer tubes, which contained EDTA and aprotinin (0.6 TIU/mL of blood). Then, plasma was stored at –70°C until the day of the assay (not exceeding 20 days). Plasma extraction and assay of UII was performed according to the instructions of the manufacturer. The standard peptide was solved in the assay buffer that contained NaH_2_PO_4_, Na_2_HPO_4_, NaCl, EDTA, bovine serum albumin (BSA), and sodium azide. A standard curve was obtained from the known concentrations of standard peptide on the log scale (*x*-axis), and its corresponding optic density (OD) reading (carried out at 450 nm) on the linear scale (*y*-axis). There was negligible difference as regards optical properties at the actual wavelength between the medium for standards and the plasma extract. The concentration of UII in a sample was determined by locating its OD on the *y*-axis, then drawing a horizontal line to intersect with the standard curve. From this point, a vertical line was drawn to intersect the *x*-axis, and the UII concentration of the sample was calculated. If necessary, samples were diluted prior to the assay, and then the measured concentration was multiplied by their respective dilution factors.

### Statistical analysis

Normally distributed data are expressed as mean ± SE. When data are not normally distributed, the median, maximum, and minimum values are also given. The normally distributed data were compared by one-way ANOVA, whereas the not-normally distributed data were compared using Kruskal–Wallis test. ANOVA was followed by Tukey’s HSD test, wherever applicable. Univariate two-way ANOVA and Mann–Whitney *U* test were used to analyze the differences in UII levels between males and females subgroups. A two-sided *P* < 0.05 was considered significant.

## Results

No differences were observed in age and gender between the groups, but the creatinine levels in group 2 differed from those in groups 1 and 3 (*P* = 0.001 and < 0.0001, respectively) (Table I). The GFRs of groups 1, 2, and 3 were 81.29 ± 17.92, 16.74 ± 4.32, and 198.36 ± 100.03 mL/min, respectively. No history of drug use, diabetes mellitus (DM), hypertension (HT), or other co-morbidities were reported in group 3. No significant difference was observed in the rate of use of calcium channel blockers (CCBs), angiotensin-converting enzyme (ACE) inhibitors, or angiotensin receptor blockers (ARBs) (alone or in combination with other antihypertensive agents) between the patients in groups 1 and 2 (*P* > 0.05) (Table I). On the other hand, all patients in group 1 (tx) were on immunosuppressive drug treatment, i.e. calcineurin inhibitor (28 patients on cyclosporine and 7 patients on tacrolimus), azathioprine (7 patients), or mycophenolate (28 patients), and prednisolone (30 patients). Calcineurin inhibitors were adjusted according to blood levels (cyclosporine and tacrolimus daily doses were 289.57 ± 129.43 and 15.57 ± 1.90 mg, respectively) and daily doses of azathioprine, mycophenolate, and prednisolone were 1–3 mg/kg, 2 g, and 5–35 mg, respectively. Duration of transplantation (group 1) was 24.0 (4.5–144.0) months. No difference in the frequencies of DM or HT was observed between the patients in groups 1 and 2 (3% versus 3%, *P* = 0.175; and 37% versus 39%, *P* = 0.219, respectively), but the combination of DM and HT was lower in group 1 than in group 2 (3% versus 42%, *P* < 0.001).

The median (min–max) values of UII were as given in Table I. When these UII concentrations were compared by Kruskal–Wallis test, the total *P* value was 0.017. When logarithmic transformation was executed regarding UII (ng/mL) levels, the log (UII × 1000) levels showed a normal distribution (15,16). These log (UII × 1000) levels were then used for further analyses. When the log (UII × 1000) levels between the groups were compared by one-way analysis of variance (ANOVA), the *P* value was 0.001 (Table II). Tukey’s HSD post-hoc analysis revealed a significant difference between the UII levels in group 1 and those in groups 2 and 3 (*P* = 0.001 and 0.017, respectively), but no significant difference was observed between the UII levels in groups 2 and 3 (*P* = 0.541).

**Table II.** Comparison of log (UII × 1000) levels between males and females.

Groups	Log (UII × 1000) levels (total)^a^	Gender	No. of cases	Log (UII × 1000) levels
				Mean ± SE
1	3.0035 ± 0.60478	Male	23	3.0686 ± 0.67444
		Female	12	2.8787 ± 0.44153
2	2.6403 ± 0.29595	Male	22	2.7468 ± 0.22989
		Female	14	2.4730 ± 0.31798
3	2.7400 ± 0.24080	Male	21	2.7607 ± 0.27365
		Female	18	2.7158 ± 0.20089

^a^One-way analysis of variance (ANOVA) test’s *P* value was 0.001 (see text).

UII = plasma urotensin II level (ng/mL); SE = standard error of the mean.

There was no correlation between age, creatinine levels, GFR, co-morbidities (DM and HT), antihypertensive drug use, or log (UII × 1000) levels among the three groups. In group 1, there was also no correlation between log (UII × 1000) levels and transplantation duration or immunosuppressive drug doses.

**Figure 1.**
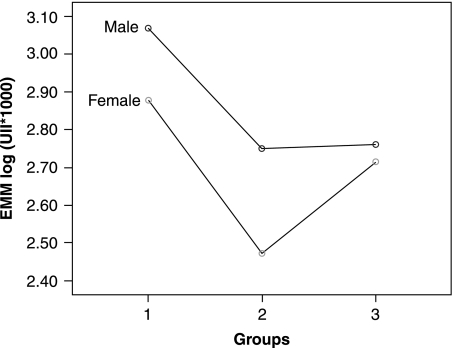
Comparison of log (UII × 1000) levels between the groups. EMM = estimated marginal means; UII = urotensin II (ng/mL).

Males in all three groups tended to have higher log (UII × 1000) levels than females (*P* = 0.039 on univariate two-way ANOVA) (Table II and Figure 1). Further analysis by Mann–Whitney *U* test, however, showed that only group 2 males log (UII × 1000) levels were significantly higher than females, while log (UII × 1000) levels of males in groups 1 and 3 were not (2.77 [2.27–3.06] versus 2.56 [1.74–2.82], 2.82 [2.26–5.27] versus 2.85[2.19–4.04], and 2.78[1.78–3.27] versus 2.70 [2.39–3.04]; median (min-max) values; *P* = 0.009, 0.578, and 0.266, respectively).

## Discussion

Some investigators implicate UII in the pathophysiology of many diseases including CKD. Previous studies have shown an increase in the UII levels in patients with CKD, including those undergoing hemodialysis (HD) (1).

Mosenkis et al. (17) compared the UII levels in three patient groups: patients on HD (group 1), patients with CKD not on HD (group 2), and subjects with normal renal function (group 3). In contrast to the results of previous studies, they found that the mean plasma UII levels (pg/mL) were highest in group 3, low in group 1, and lowest in group 2 (22445 ± 652, 16351 ± 641, and 13773 ± 652, respectively; *P* < 0.0001). The very low UII level in group 2 was attributed mostly to reduced production and simultaneous increased clearance of UII by diseased kidneys in comparison to the patients on HD.

In our study, the plasma UII levels tended to be higher in control subjects (group 3) than in patients with CKD but not on HD treatment (group 2) (Table II), but the results were not statistically significant; *post-hoc* analysis showed *P* > 0.05. The UII measurements in our study were made by enzyme-linked immunoassay, whereas the above study used radioimmunoassay. This difference in methodology may partly explain the difference in the results of these studies (1).

In Mosenkis’s study 60% of the subjects in the control group were hypertensive, and 23% were diabetic; while in our study the control group subjects were neither hypertensive nor diabetic (we aimed in our study to compare UII concentrations observed in normal physiological conditions with those observed in diseased conditions; therefore, subjects in our control group were selected from completely healthy subjects). This difference in the rate of hypertensive and diabetic subjects between the control groups of the two studies may also explain the differences in the results (1,3,18–20).

Another feature of Mosenkis’ study is the presence of African-Americans in the study population (17). African-Americans have a high prevalence of insulin resistance but paradoxically a low prevalence of metabolic syndrome (21). These ethnicity and race differences between the two study groups may also explain the differences in the results of UII between the two studies (10,21).

Interestingly, the plasma UII level was significantly higher in the tx (group 1) patients than in the other two groups in our study (Table II and Figure 1). Kidneys have a major role in UII production (5). Still this increased plasma level could not be explained solely by the transplanted kidney, because the plasma level is higher than that of the healthy controls who had two normal functioning kidneys. The tx group was on immunosuppressive medications (mostly calcineurin inhibitor, azathioprine or mycophenolate, and prednisolone) (11). The calcineurin inhibitors cyclosporine (CsA) and tacrolimus can cause HT and induce acute and chronic nephrotoxicity by different mechanisms such as vasoconstriction (through the release of different vasoactive substances) and tubulointerstitial fibrosis (22–25). Cyclosporine infusion can lead to proximal tubular damage through an increase in intracellular Ca^2+^, which is completely prevented by the calcium channel blocker nifedipine (25). Up-regulation of UII and its receptor can also cause renal fibrosis and dysfunction (4). Moreover, UII has a vasoconstrictive effect. Activation of UTR by UII increases phosphoinositide (PI) turn-over with an increase in intracellular Ca^2+^. The PI turnover and vasoconstrictive effect of UII are inhibited by phospholipase C inhibition with 2-nitro-4-carboxyphenyl-N (1,26). The calcium channel blockers verapamil, nifedipine, and diltiazem can also inhibit the vasoconstrictive effect of UII (26).

There are reports of a gender effect on the survival of patients with a transplanted kidney and on the CsA nephrotoxicity rate, even at similar daily doses. This has been attributed to differences in sex hormones between males and females (27,28). The difference in males’ and females’ log (UII × 1000) levels in group 2 of our study (Table II and Figure 1) may explain this gender difference in the rate of graft survival. The similarity in the effect of UII and the side-effects of calcineurin inhibitors, which can be partially prevented by calcium channel blockers, shows that a relationship may exist between calcineurin inhibitors and the elevated UII levels in our tx patients. None of our group 1 patients was on calcineurin inhibitor-sparing regimens (as we mentioned before, we tried to include somewhat stable and non-complicated patients in this group), so we did not have the chance to see UII levels in patients not taking calcineurin inhibitors. Comparing log (UII × 1000) levels (med [min–max]) of cyclosporine with tacrolimus-taking groups yielded no significant difference (2.82 [2.19–5.27] versus 2.89 [2.52–3.76], respectively; *P* = 0.88). But we should mention that the tacrolimus-taking group (*n* = 7) was small.

There is an international effort to standardize and improve the management of this important population of patients (11). Measuring plasma UII levels at different stages, including nephrotoxicity, rejection, and other complication states, with different immunosuppressive drug dosing and regimens may help elucidate the role of UII in the above-mentioned medical conditions and may lead to the generation of new treatment models that modulate the level and effect of UII.

## Conclusion

The high UII level in tx patients in comparison with healthy controls could be attributed to the use of mandatory immunosuppressive drugs. Further studies are needed to ascertain the beneficial and/or harmful aspects of elevated UII levels to improve management strategies for kidney recipients.
